# Intra‐patient variability of heteroplasmy levels in urinary epithelial cells in carriers of the m.3243A>G mutation

**DOI:** 10.1002/mgg3.523

**Published:** 2018-12-04

**Authors:** Paul de Laat, Richard J. Rodenburg, Jan A. M. Smeitink, Mirian C. H. Janssen

**Affiliations:** ^1^ Department of Pediatrics, Radboudumc Amalia Childrens Hospital Radboud Center for Mitochondrial Medicine Nijmegen The Netherlands; ^2^ Department of Internal Medicine, Radboudumc Radboud Center for Mitochondrial Medicine Nijmegen The Netherlands

**Keywords:** heteroplasmy, m.3243A>G, maternally inherited diabetes deafness, mitochondrial medicine, mitochondrial myopathy, encephalopathy, lactic acidosis and stroke‐like episodes, NMDAS, outcome measures

## Abstract

**Background:**

The mitochondrial DNA m.3243A>G mutation is one the most prevalent mutation causing mitochondrial disease in adult patients. Several cohort studies have used heteroplasmy levels in urinary epithelial cells (UEC) to correlate the genotype of the patients to the clinical severity. However, the interpretation of these data is hampered by a lack of knowledge on the intra‐patient variability of the heteroplasmy levels. The goal of this study was to determine the day‐to‐day variation of the heteroplasmy levels in UEC.

**Methods:**

Fifteen carriers of the m.3243A>G mutation collected five urine samples in a 14‐day window. Heteroplasmy levels of the m.3243A>G mutation were determined in these samples. Data from the national cohort study, including Newcastle Mitochondrial Disease Adult Scale scores and clinical diagnosis, were used.

**Results:**

In the samples of six patients, heteroplasmy levels were within a 5% margin. In the samples collected from five patients, the margin was >20%.

**Conclusion:**

Heteroplasmy levels of UEC in carriers of the m.3243A>G mutation have a significant day‐to‐day variation. The interpretation of a correlation between heteroplasmy levels in urine and disease severity is therefore not reliable. Therefore, heteroplasmy levels in UEC should not be used as a prognostic biomarker in these patients.

## 1 INTRODUCTION

In 1990, the Adenine to Guanine transition at position 3,243 of mitochondrial DNA (m.3243A>G) in the *MT‐TL1* encoding tRNA^LEU(UUR)^ was found as the molecular basis for MELAS (Goto, Nonaka, & Horai, 1990; Kobayashi et al., 1990). The acronym MELAS was first used in 1984 by Pavlakis, Phillips, DiMauro, De Vivo, and Rowland (1984) to describe a group of patients with mitochondrial myopathy, encephalopathy, lactic acidosis, and stroke‐like episodes. As the m.3243A>G mutation is the most common cause of MELAS syndrome (MIM 540000), it is also reported as the MELAS mutation although other phenotypic expressions have been described. These include maternally inherited diabetes and deafness (MIDD, MIM 520000) (Ouweland et al., 1992), hypertrophic cardiomyopathy (Lev et al., 2004), macular dystrophy (Laat, Smeitink, Janssen, Keunen, & Boon, 2013), gastrointestinal involvement (Laat et al., 2015),and oligosymptomatic variants of the acronym MELAS (Dvorakova et al., 2016). The m.3243A>G mutation is one the most prevalent pathogenic mutation of the mitochondrial DNA, prevalence being reported in the range of 7.59–236/100,000 persons (Chinnery et al., 2000; Majamaa et al., 1998; Manwaring et al., 2007).

Since mitochondria and mitochondrial (mt) DNA are present in all tissues except red blood cells, heteroplasmy percentages can theoretically be assessed in virtually every tissue. Two problems arise when testing heteroplasmy: Most human tissues are practically not accessible and differences in heteroplasmy levels between samples might exist. For example, invasively obtained skeletal muscle tissue DNA usually gives higher and more consistent heteroplasmy levels than DNA extracted from a less invasively obtained blood sample (Rahman, Poulton, Marchington, & Suomalainen, 2001). The lower levels in blood might even lead to false‐negative results (Laat et al., 2012). Previous studies showed a superiority of urine over blood as preferred noninvasive tissue for mutation analysis in patients at risk of carrying the m.3243A>G mutation (Frederiksen et al., 2006; Laat et al., 2012; Ma et al., 2009; Marotta et al., 2009). However, the relationship between mutation load and clinical phenotype has been a subject of research for many years (Chinnery, Howell, Lightowlers, & Turnbull, 1997; Grady et al., 2018; Liu et al., 2012; Nesbitt et al., 2013).

Surprisingly in several studies, including one of ourselves, a relationship between heteroplasmy levels in urinary epithelial cells (UEC) and clinical symptoms was suggested. In these small sample sizes, the reported correlation coefficients were however rather low (Laat et al., 2012; Ma et al., 2009; Nesbitt et al., 2013; Whittaker et al., 2009). There is little evidence on whether heteroplasmy levels (in any tissue) correlate with progression of the mitochondrial disease. A recent study showed an association between disease progression and age‐adjusted heteroplasmy in blood (Grady et al., 2018). In the other mentioned cohort studies, UEC's have predominantly been investigated as a prognostic marker for severity of disease and disease progression. In this study, we show that the measurement of m.3243A>G UEC heteroplasmy levels have a large intra‐patient day‐to‐day variability. Cautiousness regarding the usage of m.3243A>G heteroplasmy levels in UEC as a prognostic biomarker as, for example, in drug intervention studies is warranted.

## 2 METHODS

### 2.1 Patients

All subjects were genetically diagnosed with the m.3243A>G mutation in DNA extracted from skeletal muscle and/or blood. They all participate in our natural history cohort study (Laat et al., 2012). The ethics committee of the Nijmegen‐Arnhem region approved this study. Written informed consent according to the Helsinki agreement was obtained from all patients.

Patient characteristics regarding age, sex, and clinical expression of the m.3243A>G were extracted from the data of the national cohort study, including Newcastle Mitochondrial Disease Adult Scale (NMDAS)‐scores, and mtDNA heteroplasmy levels in other tissues. All patients were asked to report symptoms of urinary tract infections, fever, smoking, and alcohol use.

### 2.2 Urine sample collection and mutation analysis

All patients received an isolation box with 5 urine containers. They were instructed to collect five urine samples in a 14‐day window. The urine samples were to be collected in the morning of days 1, 4, 7, 10, and 13. The samples were stored at 3–6°C and send with regular postal service to the laboratory in the provided isolation box after collection of the fifth sample. DNA was isolated from the urine samples, after centrifugation of the urine for 10 min at 3,000 rpm, and the pellet was washed with phosphate‐buffered saline. DNA was extracted using a commercially available DNA isolation kit (PuregeneTM DNA isolation kit; Gentra Systems, MN).

Heteroplasmy levels were determined in all urine samples using PyrosequencingTM technology (Pyrosequencing, Uppsala, Sweden) as earlier described by Lowik, Hol, Steenbergen, Wetzels, and van den Heuvel (2005). The pyrosequencing reaction of the m.3234A>G mutation had a precision of 1.5%, and the lowest limit of detection was 5%. The detection limit for the m.3243A>G mutation was determined by serial dilutions of a sample containing this mutation with wild‐type mtDNA.

### 2.3 Statistics

We used descriptive statistics in analyzing the data.

## 3 RESULTS

### 3.1 General patient characteristics

Fifteen carriers were included in the study (Table 1). Four carriers (27%) were male. Median age was 39 years (range: 20–69 years). Patients had different phenotypic expressions of the m.3243A>G mutation: MELAS syndrome (one patient), MIDD (seven patients), isolated myopathy and fatigue (four patients), and cardiomyopathy (one patient). The remaining two patients were clinically asymptomatic and should be categorized as dormant carriers. The median NMDAS score was 8 (range: 1–56 with 1 being the least severe disease expression).

**Table 1:** Heteroplasmy levels of the m.3243A>G mutation in UEC's on 5 different days in a 2 week period

No.	Sex/age (years)	Clinical diagnosis	NMDAS	Intra‐patient variability (UEC; %)					Previous samples (%)		
				Day 1	Day 4	Day 7	Day 10	Day 13	Urinary epithelial cells	Blood	Saliva
1	M/33	MELAS	56	98	97	97	97	97	96	49	63
2	M/23	Myopathy	8	96	95	na	96	95	96	49	68
3	M/69	MIDD	21	87	86	84	83	82	86	11	41
4	M/50	MIDD	7	74	na	75	76	77	75	19	33
5	F/35	Cardiomyopathy	16	72	88	85	64	66	72	42	47
6	F/20	Dormant carrier	1	60	56	74	54	72	74	39	55
7	F/42	MIDD	11	75	51	60	56	72	73	29	50
8	F/38	Myopathy	3	55	63	62	69	58	55	11	na
9	F/39	MIDD	7	62	42	56	53	54	61	21	40
10	F/48	MIDD	11	43	42	47	40	45	40	23	42
11	F/61	Dormant carrier	3	43	36	39	29	40	38	7	16
12	F/34	MIDD	8	35	34	34	40	41	40	27	45
13	F/67	Myopathy	11	31	20	44	17	21	15	5	25
14	F/36	Myopathy	2	15	13	15	15	13	22	8	10
15	F/65	MIDD	18	6	6	4	2	6	6	5	23

Notes

MIDD, maternally inherited diabetes and deafness; na: not available; NMDAS: Newcastle Mitochondrial Disease Adult Scale.

### 3.2 Heteroplasmy levels

**Figure 1:**
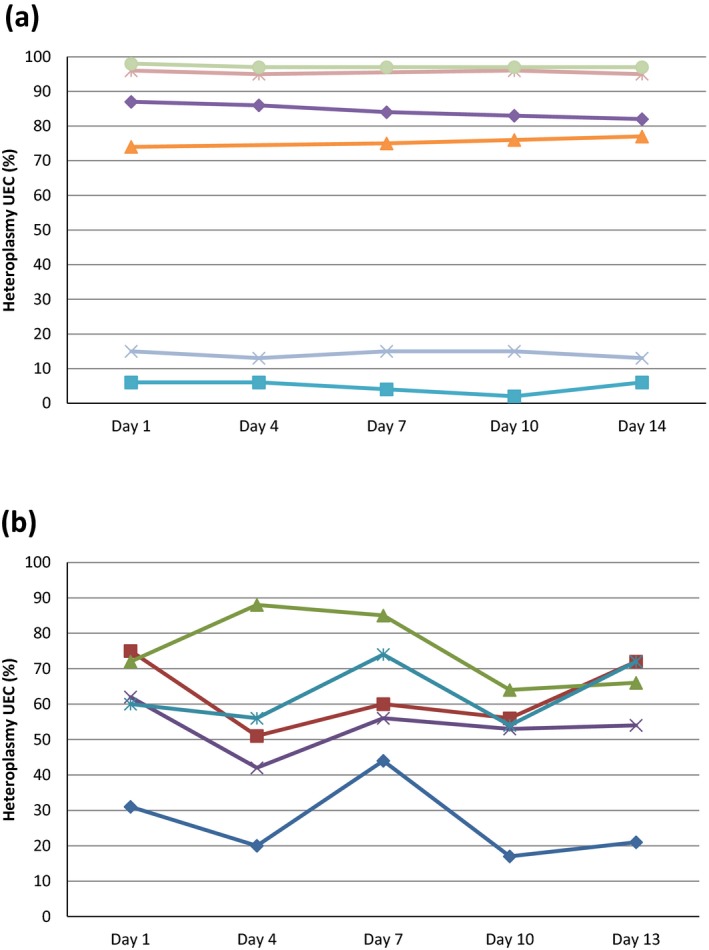
Every patient is represented by one line. (a) Heteroplasmy levels differ <5% between the different measurements. (b) Heteroplasmy levels differ >20%

A total of 75 urine samples were collected. In two samples, it was not possible to extract sufficient amounts of DNA for heteroplasmy analysis. Heteroplasmy level measurement was successful in the remaining 73 samples (Table 1). In the samples of six patients (Patients: 1, 2, 3, 4, 14, and 15), heteroplasmy levels were within 5% margin of each other (Figure 1a). In the samples of five patients (Patients: 5,6,7,9, and 13), the margin was >20% (Figure 1b). In the remaining four patients (patients: 8, 10, 11, and 12), the variation between the heteroplasmy levels in the different samples was between 5% and 20%.

Patients 9 and 12 reported some complaints of a viral upper airway infection during the first days. Patient 14 reported to have smoked during all days. There was no alcohol usage among the patients.

## 4 DISCUSSION

Urinary epithelial cells have been identified as the most optimal noninvasive tissue for measurement of heteroplasmy of mtDNA mutations (Laat et al., 2012). Heteroplasmy levels in UEC have also been correlated with disease severity (Whittaker et al., 2009). In this study, we show that UEC m.3243A>G heteroplasmy levels within one patient might show important day‐to‐day variations. Based on these results, we conclude that the level of UEC heteroplasmy should be cautiously interpreted in predicting disease severity and the results of intervention studies in carriers of the m.3243A>G mutation.

We hypothesize that the difference in heteroplasmy levels between the different urine samples from individual patients is caused by a variation in different types of (epithelial) cells in the urine samples. Previous studies extensively studied the different epithelial cells in random urine samples and report a broad variation in different kinds of epithelial cells between samples (Schumann, 1981). Due to a genetic bottleneck, there is variation between the heteroplasmy levels of cells from different organs or tissues in one patient (Cree, Samuels, & Chinnery, 2009). If there is a larger proportion of the cells in a urine sample with a higher or lower heteroplasmy level compared to another sample, this could explain the differences reported in this study.

Patients with a very high or very low heteroplasmy level in UEC have a smaller range of heteroplasmy levels compared to patients with heteroplasmy levels in the middle range (Figure 1a,b). This is consistent with our hypothesis that there is a variation in cell types in the different urine samples, a normal distribution of the variance can be expected.

As several cohort studies (Laat et al., 2012; Nesbitt et al., 2013), including one of our own, have used the heteroplasmy levels in UEC to correlate the genotype of the patients to the clinical severity, it is essential to have studied the intra‐patient variability of the heteroplasmy levels in UEC. Overall, the disease progression in carriers of the m.3243A>G mutation has no significant day‐to‐day variation. The interpretation of a correlation between heteroplasmy levels in urine and disease severity is therefore not reliable.

## 5 CONCLUSION

In this study, we demonstrated that the measurement of m.3243A>G heteroplasmy levels in UEC might have a substantial intra‐patient day‐to‐day variability. The use of UEC m.32343A>G heteroplasmy levels as a prognostic biomarker should be interpreted in light of these findings.

## DISCLOSURE

Jan Smeitink is the founding CEO of Khondrion BV.

## AUTHORS’ CONTRIBUTIONS

Conception and design: PdL, MJ, JS; Analysis and interpretation of data: PdL, RR, MJ; Drafting the article: PdL; Critically revising the manuscript: RR, MJ, JS.
